# Supplementary material for: Microbiome of Nodules and Roots of Soybean and Common Bean: Searching for Differences Associated with Contrasting Performances in Symbiotic Nitrogen Fixation
Source: Int J Mol Sci. 2022 Oct 10;23(19):12035. doi: 10.3390/ijms231912035 (PMC9570480; doi:10.3390/ijms231912035)
Supplement: Supplementary file 1 [file ijms-23-12035-s001.zip › ijms-1857512-supplementary.pdf]

International Journal of Molecular Sciences

Microbiome of Nodules and Roots of Soybean and Common Bean: Searching for Differences Associated with Contrasting Performances in Symbiotic Nitrogen Fixation

Flávia Raquel Bender[1,2], Leonardo Cardoso Alves[3], João Fernando Marques da Silva[3], Renan Augusto Ribeiro[4], Giuliano Pauli[3], Marco Antonio Nogueira[2,4], Mariangela Hungria[1,2,4,*]

[1]*Department of Biotechnology, Universidade Estadual de Londrina, C.P. 10011, Londrina 86057-970, PR, Brazil*
[2]*Soil Biotechnology Laboratory, Embrapa Soja, C.P. 4006, Londrina 86085-981, PR, Brazil*
[3]*SuperBac Biotechnology Solutions, Estr. Vitória de São Pedro, 685, Mandaguari 86975-000, PR, Brazil*
[4]*CNPq, SHIS QI 1 Conjunto B, Blocos A, B, C e D, Lago Sul, Brasília 71605-001, DF, Brazil*
**Corresponding author: mariangela.hungria@embrapa.br; biotecnologia.solo@hotmail.com*

Supplementary Material

Table S1. Localization, geographic coordinates, altitudes and climatic characteristics of the sites where the soybean experiment was performed and of soil source for the common bean experiment.

Experiment	Site	State	Coordinates [1]	Altitude (m) [2]	Climate Type [3]	Average annual lower temperature (ºC)	Average annual higher temperature (ºC)	Average annual rainfall (mm)
Soybean	Lutécia	São Paulo	22º2’S 50º25’W	457	*Cfa*	18.3	28.3	1,314
Common bean	Ponta Grossa	Paraná	25°13’S 50°10’W	880	*Cfb*	8.4	25.9	1,507

[1] Latitude, longitude
[2] Above sea level
[3] According to Köppen-Geiger classification

Table S2. Chemical properties and granulometric fractions (0 – 20 cm), and population of compatible soybean (Lutécia) or common bean (Ponta Grossa) rhizobia (0 – 10 cm) in the soils where plants were grown.

Site	Chemical properties										Rhizobia	Granulometry		
	C	pH	P	H+Al	Al	K	Ca+Mg	CEC[b]	SB	V		Clay	Silt	Sand
	g dm^{-3}	$CaCl_2$	mg dm^{-3}	-------------------------		$cmol_c$ dm^{-3}	--------------------------------			%	cells g^{-1}	------------ g kg^{-1} ---------		
Lutécia	23.00	5.60	18.99	1.60	0.00	2.60	4.90	9.10	7.50	82.41	7.6×10^1	119	11	870
Ponta Grossa	21.70	5.68	2.55	3.63	0.00	0.11	4.55	8.29	4.66	56.21	4.6×10^4	238	30	732

Table S3. Results of the relative abundance (%) of the genera present in the microbiome of nodules of soybean non-inoculated (control), inoculated with *Bradyrhizobium* spp., or co-inoculated adding *Azospirillum brasilense.*

Genus	Soybean Nodules																	
	Control						Inoculated						Co-inoculated					
	R1	R2	R3	R4	R5	R6	R1	R2	R3	R4	R5	R6	R1	R2	R3	R4	R5	R6
Bradyrhizobium	97.76	99.62	98.36	98.52	99.93	91.55	99.30	99.16	99.77	99.09	99.58	98.33	96.33	98.18	97.55	92.25	94.55	96.45
Streptomyces	-	-	0.11	0.16	-	1.23	0.10	-	-	0.08	-	0.11	0.20	-	0.23	0.28	0.22	0.41
Niastella	-	-	-	0.04	-	-	-	-	-	-	-	-	-	-	-	-	-	-
Sphingomonas	-	-	-	0.20	-	-	-	-	-	-	-	0.05	0.23	-	-	0.23	0.08	-
Massilia	-	-	-	-	-	-	-	-	-	-	-	-	-	0.05	-	-	0.04	-
Allorhizobium – Neorhizobium - Pararhizobium - Rhizobium	-	-	0.04	0.05	-	1.75	-	-	-	-	-	-	0.22	-	0.21	0.30	0.12	0.22
Burkholderia – Caballeronia - Paraburkholderia	-	-	-	-	-	-	-	-	-	-	-	-	0.07	-	-	-	0.03	-
Bacillus	-	-	-	0.03	-	0.09	-	-	-	-	-	-	-	-	0.03	-	0.07	0.05
Novosphingobium	0.06	-	-	-	-	0.46	-	-	-	-	-	-	-	0.15	-	0.33	-	0.12
Phenylobacterium	-	-	-	-	-	-	-	-	-	-	-	-	0.05	-	-	-	0.07	-
Mycobacterium	-	-	0.05	-	-	-	0.04	-	-	-	-	-	-	-	-	0.11	-	-
Mesorhizobium	-	-	-	-	-	-	-	-	-	-	-	0.04	-	0.14	-	0.15	0.09	-
Dongia	-	-	0.01	0.04	-	-	-	-	-	0.04	-	-	0.11	-	0.09	0.07	-	-
Methylobacterium	-	-	-	-	-	-	-	0.02	-	-	-	-	0.11	0.10	0.07	0.08	0.08	-
Dyella	-	-	0.05	0.18	-	-	-	-	-	-	-	-	-	-	0.03	0.07	-	-
Amycolatopsis	-	-	-	-	-	-	-	-	-	-	0.02	-	-	-	0.03	-	0.10	0.07
Actinoplanes	-	-	-	-	-	-	-	-	-	-	-	-	0.02	-	-	-	-	-
Terriglobus	-	-	0.06	0.17	-	-	-	-	-	0.05	-	-	-	0.08	0.05	0.07	0.08	-
Chitinophaga	-	-	-	0.13	-	-	-	-	-	-	0.04	0.02	-	0.07	-	0.13	-	-
Promicromonospora	-	-	-	-	-	-	-	0.04	-	-	-	-	-	-	-	-	-	-
Candidatus_Udaeobacter	-	-	-	-	0.03	-	-	-	-	-	-	-	-	-	-	-	-	-
Caulobacter	-	-	-	-	-	-	-	-	-	-	-	-	0.04	0.12	0.11	-	-	-
Devosia	-	-	-	-	-	-	-	-	-	-	-	-	-	-	-	0.03	-	-
Mitsuaria	-	-	-	-	-	-	-	0.03	-	0.04	-	-	-	-	-	0.38	-	-

Enterobacter	-	-	-	-	-	0.11		-	-	-	-	-	-		0.40	-	0.05	-	-	-
Pseudomonas	0.16	0.06	0.02	0.04	-	0.21		-	0.02	0.04	0.07	0.11	0.03		0.06	0.08	-	0.39	0.04	0.09
Asticcacaulis	-	-	-	-	-	-		-	-	-	0.01	-	-		-	-	-	-	-	-
Lysobacter	-	-	-	-	-	-		-	-	-	-	-	-		-	-	0.02	-	-	-
Dyadobacter	0.09	-	-	-	-	-		-	-	-	-	-	-		-	-	-	0.13	-	-
Cupriavidus	-	-	-	-	-	-		-	-	-	-	-	-		-	-	-	0.39	-	-
Stenotrophomonas	0.14	-	-	-	-	0.70		-	-	-	-	-	-		-	-	-	-	-	-
Labrys	-	-	-	-	-	-		-	-	-	0.03	-	-		-	-	-	-	-	-
Acidipila	-	-	0.01	-	-	-		-	-	-	-	-	-		-	-	-	-	-	-
Comamonas	-	-	-	-	-	0.43		-	-	-	-	-	-		-	-	-	-	-	-
Mucilaginibacter	-	-	-	-	-	-		-	-	-	0.02	-	-		-	-	0.02	-	-	-
Chryseobacterium	-	-	-	-	-	0.58		-	-	-	-	-	-		-	-	-	0.57	-	-
Rhodoplanes	-	-	-	-	-	-		-	-	-	-	-	-		-	-	-	0.03	-	-
Xylophilus	-	-	-	-	-	-		-	-	-	-	-	-		0.06	-	-	-	-	-
Pseudorhodoferax	-	-	-	-	-	-		-	-	-	-	-	-		-	-	-	0.05	-	-
Escherichia - Shigella	-	-	0.01	0.05	-	-		-	-	-	0.01	0.01	0.02		-	-	-	0.03	0.05	-
Paenarthrobacter	-	-	-	-	-	-		-	-	-	-	-	-		0.03	-	-	-	-	-
Sphingobacterium	-	-	-	-	-	0.62		-	-	-	-	-	-		0.30	-	-	-	-	-
Psychrobacter	-	0.06	-	-	-	-		-	-	-	-	-	-		-	-	-	-	-	-
Methylophilus	-	-	-	-	-	-		-	-	-	-	-	-		0.06	-	-	-	-	-
Flavobacterium	-	-	-	-	-	-		-	-	-	-	-	-		0.21	-	-	-	-	-
Kluyvera	-	-	-	-	-	-		-	-	-	-	-	-		-	-	-	0.48	-	-
Paenibacillus	-	-	-	-	-	-		-	-	-	-	-	-		-	-	-	0.03	-	-
Pseudosphingobacterium	-	-	-	-	-	-		-	0.02	-	-	-	-		-	-	-	-	-	-
Kribbella	-	-	-	-	-	-		-	-	-	0.004	-	-		-	-	-	-	0.01	-

Table S4. Relative abundance (%) of bacteria identified at the phylum, class, family and/or order level, and of unassigned genera present in the microbiome of nodules of soybean non-inoculated (control), inoculated with *Bradyrhizobium* spp., or co-inoculated adding *Azospirillum brasilense*.

Genus	Soybean Nodules																	
	Control						Inoculated						Co-inoculated					
	R1	R2	R3	R4	R5	R6	R1	R2	R3	R4	R5	R6	R1	R2	R3	R4	R5	R6
UnC Chitinophagaceae	-	-	-	-	-	-	-	-	-	0.03	-	-	-	-	-	-	-	-
UnC Gammaproteobacteria I.S.	-	-	0.02	-	-	-	-	-	-	0.03	-	-	-	-	-	-	-	-
UnC Saccharimonadales	-	-	-	-	-	-	-	-	-	-	-	-	-	0.06	-	0.03	0.03	-
UnC Micropepsaceae	-	-	-	-	-	-	-	-	-	-	-	-	-	0.06	0.05	-	-	-
UnC Acidobacteriia	0.05	-	-	-	-	-	-	-	-	-	-	-	-	-	-	-	-	-
UnC Acidobacteria	-	-	-	-	-	-	-	-	-	-	-	0.01	-	-	-	-	-	-
UnC Thermodesulfovibrionia	0.02	-	-	-	-	-	-	-	-	-	-	-	-	-	-	-	-	-
Unassigned	1.64	0.05	0.30	0.22	-	1.41	0.16	0.23	-	0.11	0.07	0.04	0.47	0.42	0.43	2.14	0.59	0.86

Table S5. Relative abundance (%) of genera present in the microbiome of roots of soybean non-inoculated (control), inoculated with *Bradyrhizobium* spp., or co-inoculated adding *Azospirillum brasilense*.

Genus	Soybean Roots																	
	Control						Inoculated						Co-inoculated					
	R1	R2	R3	R4	R5	R6	R1	R2	R3	R4	R5	R6	R1	R2	R3	R4	R5	R6
Bradyrhizobium	4.59	10.51	8.64	4.41	9.40	5.61	13.49	11.31	18.14	10.08	8.50	5.89	41.00	11.37	37.55	9.92	12.08	8.66
Streptomyces	19.07	16.99	17.69	19.85	20.07	6.70	17.13	18.66	14.42	19.47	15.33	7.57	6.24	13.46	10.25	19.26	17.83	23.20
Niastella	5.83	5.83	3.90	2.82	8.41	2.89	5.17	3.32	7.62	5.70	8.89	7.81	3.34	7.35	4.21	6.38	4.10	4.62
Sphingomonas	2.45	4.10	3.69	3.79	3.67	2.18	3.27	3.08	3.74	3.91	3.75	7.53	2.66	3.16	2.87	3.34	2.98	4.05
Massilia	2.03	4.36	1.58	2.17	3.63	1.51	1.75	1.59	2.05	2.45	5.74	4.15	2.00	5.67	2.54	3.95	11.17	6.10
Allorhizobium - Neorhizobium - Pararhizobium - Rhizobium	3,39	4,05	5,31	3,47	4,10	3,68	6,20	5,13	4,62	4,85	2,78	3,77	2,92	4,69	4,12	4,35	3,87	5,30
Burkholderia – Caballeronia - Paraburkholderia	2.07	2.66	1.84	1.48	1.99	13.65	0.80	1.42	0.89	1.39	1.91	12.83	1.21	1.99	0.94	1.81	2.91	2.43
Bacillus	3.38	4.70	3.80	3.63	1.19	1.20	5.72	2.40	3.27	3.19	2.33	0.90	0.32	2.52	2.44	3.42	1.18	1.35
Novosphingobium	1.75	1.97	3.95	1.68	1.93	1.44	2.38	1.92	1.57	1.87	1.02	1.59	2.01	2.05	2.13	2.38	0.97	1.74
Phenylobacterium	1.28	1.47	2.29	1.48	1.80	2.48	0.99	1.39	1.79	1.73	1.39	2.45	0.85	1.58	0.96	1.57	2.13	2.63
Mycobacterium	1.18	1.72	2.70	2.26	2.08	2.00	1.90	2.64	1.42	1.68	1.75	0.90	0.50	0.42	0.58	1.80	1.91	2.60
Mesorhizobium	2.04	1.87	2.12	2.14	2.05	0.98	1.95	1.86	1.65	2.11	1.48	1.60	0.96	1.76	0.98	1.42	1.40	1.22
Dongia	1.43	1.59	3.30	1.19	1.93	0.30	1.78	1.42	1.62	1.38	1.59	0.68	0.80	1.20	0.92	0.66	0.36	0.49
Streptacidiphilus	0.87	1.44	-	0.84	1.07	1.31	1.60	1.29	1.50	1.43	2.30	1.79	0.38	0.87	0.82	1.52	2.26	1.15
Methylobacterium	0.78	0.62	0.75	1.09	1.50	1.30	1.15	1.34	1.21	1.47	1.44	0.45	0.56	1.02	0.81	1.65	1.69	1.91
Dyella	0.22	-	1.12	0.84	0.63	8.89	0.84	0.57	0.24	0.41	0.29	3.81	0.27	0.55	0.59	0.61	0.14	0.47
Amycolatopsis	0.85	-	0.53	0.88	1.44	1.61	0.88	1.18	1.04	1.09	1.26	1.24	0.19	0.98	0.55	0.83	2.34	2.28
Actinoplanes	1.21	0.61	2.26	1.57	0.87	0.67	1.48	1.00	0.85	0.86	1.10	0.95	0.71	0.23	1.07	1.45	1.14	0.94
Terriglobus	0.56	0.94	1.05	0.81	1.86	1.11	1.13	1.10	0.81	0.84	1.20	1.10	0.43	0.76	0.49	1.39	1.19	1.03
Chitinophaga	0.75	1.37	1.00	1.76	0.91	0.82	1.31	1.56	0.59	0.41	0.77	1.09	0.53	1.58	0.68	0.86	0.36	0.62
Promicromonospora	0.88	0.81	2.25	1.61	1.06	2.40	1.02	1.26	0.58	0.53	-	-	0.23	0.15	0.37	0.55	-	-
Candidatus_*Udaeobacter*	0.87	1.42	1.08	1.15	0.54	0.58	0.95	0.82	0.91	0.92	1.22	0.71	0.29	0.29	0.52	0.30	0.36	0.34
Caulobacter	0.81	1.67	1.38	0.83	0.62	0.49	0.64	1.18	0.43	0.82	0.70	0.41	0.34	0.29	0.51	0.72	0.44	0.53
Devosia	0.68	0.66	0.77	0.75	0.43	0.85	0.95	0.40	0.56	0.60	0.43	0.60	0.40	0.39	0.56	0.52	0.23	0.90
Mitsuaria	0.63	1.01	0.50	0.36	0.38	-	1.19	-	-	0.35	0.40	0.48	0.29	0.53	0.49	0.35	0.44	0.69
Edaphobacter	0.24	0.75	1.16	0.58	0.48	0.65	0.42	0.69	0.62	0.43	0.32	0.46	0.15	0.39	-	0.49	0.23	0.28

Variovorax	0.52	0.55	0.62	0.45	0.48	0.23		0.67	-	0.34	0.54	0.49	0.47		0.32	0.69	0.40	0.45	0.41	0.32
Enterobacter	-	0.11	-	-	-	-		0.39	0.27	-	0.07	-	-		5.70	-	0.19	-	-	0.13
Hyphomicrobium	0.33	0.42	0.64	0.36	0.39	-		0.80	0.53	0.40	0.50	0.35	0.35		0.27	0.31	0.37	0.36	0.21	0.42
Pseudomonas	0.22	0.36	-	0.10	0.19	0.18		0.45	0.34	0.08	0.28	0.27	0.15		0.21	0.47	0.35	0.52	0.38	0.51
Lechevalieria	0.64	0.59	0.65	0.25	0.53	-		-	0.72	-	0.88	0.49	-		0.18	-	-	-	0.42	0.58
Pseudoduganella	0.21	1.09	-	0.28	-	-		-	0.59	0.53	0.45	-	0.62		0.45	1.06	-	0.52	-	-
Actinomadura	0.22	0.24	0.42	0.60	0.49	-		-	0.48	0.33	0.47	0.37	0.44		-	0.19	-	0.31	0.69	0.23
Asticcacaulis	0.55	0.52	0.26	0.21	0.39	0.10		-	0.29	0.49	0.20	0.48	0.48		0.26	0.27	0.28	0.22	0.20	0.15
Lysobacter	0.26	0.33	0.40	0.55	0.13	0.62		-	0.30	0.20	0.33	0.16	0.46		0.29	0.28	0.23	0.17	0.13	0.24
Dyadobacter	0.38	0.10	0.13	0.27	0.16	0.40		-	0.61	0.18	-	-	0.19		0.24	0.26	0.17	0.51	-	0.16
Cupriavidus	0.24	-	-	0.10	0.31	0.17		-	-	0.28	0.29	0.39	0.44		0.29	0.42	-	0.27	0.34	-
Herbiconiux	0.11	-	-	0.14	0.35	0.29		0.94	0.64	-	0.53	0.32	-		-	-	-	-	-	0.58
Stenotrophomonas	0.10	-	0.13	0.16	-	0.38		-	0.51	0.13	-	-	-		0.73	0.15	0.27	0.14	-	0.14
Labrys	0.16	-	0.50	0.39	0.24	0.21		1.16	-	-	-	0.34	-		0.10	-	-	0.15	0.16	-
Parafilimonas	0.21	0.24	0.19	0.17	-	-		0.34	0.29	0.32	0.20	0.56	0.22		0.10	-	-	0.22	-	0.16
Acidipila	0.08	0.18	0.69	0.31	0.07	1.06		-	0.13	0.25	0.11	-	0.08		-	-	-	-	-	0.11
Kutzneria	-	0.22	-	0.10	0.13	1.43		-	-	0.24	-	0.23	0.60		-	-	-	-	-	-
Dactylosporangium	0.42	0.30	0.26	0.25	0.13	-		-	0.32	0.29	0.24	-	-		0.15	0.23	0.23	-	0.09	-
Bryobacter	0.05	-	0.20	0.28	-	0.47		-	-	-	-	0.36	0.72		0.12	0.14	-	-	0.25	0.29
Kribbella	0.12	0.14	-	0.40	0.41	0.37		-	-	-	0.24	0.23	0.20		0.04	-	-	-	0.37	0.30
Dokdonella	0.35	-	0.47	0.35	0.25	0.08		-	0.21	-	-	0.27	-		0.16	0.29	-	0.18	0.10	0.12
Comamonas	0.22	-	-	0.19	-	-		-	-	0.33	0.53	-	-		-	0.39	-	-	0.31	-
Actinophytocola	0.09	-	-	0.59	-	-		0.77	-	0.22	-	-	0.28		-	0.37	-	-	-	-
Mucilaginibacter	0.19	-	0.33	0.25	0.16	0.24		-	-	-	0.18	0.15	-		0.14	0.04	-	0.19	0.11	0.18
Chryseobacterium	0.19	-	-	0.09	-	-		-	-	-	-	-	-		-	-	0.24	0.46	-	-
Actinospica	-	-	-	0.13	0.19	0.26		-	0.28	-	0.13	0.22	-		0.06	0.17	0.11	0.22	0.15	0.19
Aridibacter	0.14	0.28	0.23	0.11	0.11	0.04		0.19	0.11	0.12	0.12	-	-		0.11	0.10	0.10	0.15	-	0.09
Methylopila	0.13	-	0.27	0.37	0.13	0.25		-	0.34	0.16	-	-	-		0.13	-	-	-	-	0.17
Inquilinus	0.12	0.13	0.17	0.54	0.08	0.24		-	0.20	-	0.07	-	-		-	0.14	0.06	0.05	-	-
Rhodoplanes	0.20	-	-	0.17	-	-		-	-	-	0.41	0.38	-		-	-	0.49	-	-	-
Pseudolabrys	0.07	-	-	0.12	-	-		-	-	0.63	-	0.46	-		-	-	-	-	0.42	-
Xylophilus	0.15	-	0.26	0.15	-	-		-	0.67	0.14	-	-	-		0.24	-	-	-	-	-
Pseudorhodoferax	0.13	-	-	0.14	-	0.19		0.50	0.23	-	0.14	-	-		0.14	-	-	-	-	-

Escherichia-Shigella	0.10	-	0.10	0.09	-	0.08	-	0.08	0.11	-	0.14	0.18	0.10	0.15	-	0.08	0.06	-
Chthoniobacter	0.12	0.15	0.24	0.17	0.22	-	-	-	-	0.16	-	-	-	-	-	0.15	0.11	0.12
Steroidobacter	0.08	0.15	-	0.10	0.15	0.33	-	-	-	0.16	0.17	-	0.05	0.09	-	0.13	-	-
Sphaerisporangium	1.36	-	-	-	-	-	-	-	-	-	-	-	-	-	-	-	-	-
Curtobacterium	-	-	-	-	-	-	-	-	-	-	-	-	-	-	0.42	0.50	0.33	-
Nordella	0.12	-	0.19	0.10	0.06	-	-	-	0.10	0.10	0.14	-	0.09	0.13	0.07	0.08	-	-
Paenarthrobacter	0.04	0.30	-	0.05	-	-	0.23	-	-	-	0.12	-	0.17	-	-	0.22	-	-
Sphingobacterium	-	-	-	-	-	-	-	-	-	-	-	-	0.20	-	-	-	-	-
Nocardioides	0.12	-	-	0.53	0.03	-	0.31	-	-	-	-	-	-	-	-	0.08	-	0.05
Rhizobacter	0.35	-	0.51	0.15	-	-	-	-	-	-	-	-	-	-	-	-	-	-
Ralstonia	0.04	-	-	-	-	-	-	-	-	-	-	0.95	-	-	-	-	-	-
Pelomonas	0.16	-	-	0.10	-	-	-	-	0.50	-	-	-	0.22	-	-	-	-	-
Candidatus_*Solibacter*	-	-	-	0.35	0.25	-	-	-	-	0.26	-	-	-	-	-	-	-	-
Terrabacter	0.12	-	-	-	0.31	-	-	-	-	0.42	-	-	-	-	-	-	-	-
Marmoricola	0.12	-	0.26	0.09	-	-	-	-	0.17	-	-	-	-	-	-	0.17	-	-
Roseateles	-	-	-	-	-	-	-	-	-	-	-	-	0.20	-	-	0.60	-	-
Reyranella	-	-	-	0.26	0.28	-	-	-	-	0.24	-	-	-	-	-	-	-	-
Lysinimonas	0.13	-	0.64	-	-	-	-	-	-	-	-	-	-	-	-	-	-	-
Rhodopseudomonas	0.12	-	0.20	0.18	-	0.24	-	-	-	-	-	-	-	-	-	-	-	-
Psychrobacter	-	-	-	-	-	-	-	0.25	-	-	-	-	-	-	-	0.37	-	-
Micromonospora	-	-	-	-	-	-	-	-	-	-	-	-	-	-	-	-	-	0.43
Methylophilus	-	-	-	-	-	0.12	-	-	-	0.17	-	-	0.14	0.14	-	-	-	-
Terrimicrobium	0.15	-	-	0.12	-	-	-	0.10	0.11	-	-	0.06	-	0.08	-	-	-	-
Leifsonia	-	-	-	0.08	-	0.32	-	-	-	-	-	0.21	-	-	-	-	-	-
Flavobacterium	0.06	-	-	0.12	-	-	-	-	0.08	-	-	-	0.06	-	-	-	-	-
Microvirga	0.05	0.23	-	0.10	0.09	-	-	-	-	-	-	-	-	-	-	-	-	-
Leptothrix	-	-	-	-	-	-	-	-	-	-	-	-	-	-	-	-	0.47	-
Pseudonocardia	0.10	-	-	0.30	-	-	-	-	-	-	-	-	-	-	-	-	-	0.05
Granulicella	-	-	-	-	-	0.44	-	-	-	-	-	-	-	-	-	-	-	-
Solirubrobacter	0.19	-	-	0.24	-	-	-	-	-	-	-	-	-	-	-	-	-	-
Aeromicrobium	-	-	-	0.10	-	-	-	0.33	-	-	-	-	-	-	-	-	-	-
Catenulispora	-	-	-	-	-	0.36	-	-	-	-	0.03	-	-	-	-	-	-	-
Flavisolibacter	0.06	-	-	-	-	-	-	0.12	-	-	-	0.12	-	-	-	-	0.06	-

Pedomicrobium	0.04	-	-	0.09	-	-	-	0.16	-	-	-	-	-	-	-	-	-	-
Jatrophihabitans	0.12	-	-	0.16	-	-	-	-	-	-	-	-	-	-	-	-	-	-
Shinella	-	-	-	-	-	0.28	-	-	-	-	-	-	-	-	-	-	-	-
Brevundimonas	-	-	-	-	-	-	-	-	-	0.11	-	-	-	-	-	-	-	0.16
Pseudoxanthomonas	-	0.11	-	-	-	-	-	-	-	-	-	-	0.16	-	-	-	-	-
Pantoea	-	-	-	0.26	-	-	-	-	-	-	-	-	-	-	-	-	-	-
Kitasatospora	0.22	-	-	-	-	-	-	-	-	-	-	-	-	-	-	-	-	-
Sorangium	-	-	-	0.21	-	-	-	-	-	-	-	-	-	-	-	-	-	-
Bosea	0.10	-	-	0.10	-	-	-	-	-	-	-	-	-	-	-	-	-	-
Acidothermus	-	-	-	0.12	-	-	0.07	-	-	-	-	-	-	-	-	-	-	-
Couchioplanes	0.17	-	-	-	-	-	-	-	-	-	-	-	-	-	-	-	-	-
Occallatibacter	0.16	-	0.26	0.19	0.25	0.52	-	-	0,26	0,14	0.13	0.58	0.10	-	-	-	0.17	0.20
Achromobacter	-	-	0.04	-	-	-	-	-	-	-	-	-	-	-	-	-	-	0.11
Actinomycetospora	0.05	-	-	0.10	-	-	-	-	-	-	-	-	-	-	-	-	-	-
Kibdelosporangium	-	-	-	0.15	-	-	-	-	-	-	-	-	-	-	-	-	-	-
Intrasporangium	-	-	-	0.15	-	-	-	-	-	-	-	-	-	-	-	-	-	-
Haliangium	0.14	-	-	0.01	-	-	-	-	-	-	-	-	-	-	-	-	-	-
Ensifer	0.08	-	-	0.15	-	-	-	-	-	-	-	-	-	-	-	-	-	-
Quadrisphaera	-	-	-	0.14	-	-	-	-	-	-	-	-	-	-	-	-	-	-
Sphingobium	0.09	-	-	0.05	-	-	-	-	-	-	-	-	-	-	-	-	-	-
Blastocatella	-	-	-	-	-	-	-	-	-	-	-	-	-	-	0.12	-	-	-
Luteolibacter	0.05	-	-	0.07	-	-	-	-	-	-	-	-	-	-	-	-	-	-
Gemmata	0.05	-	-	0.07	-	-	-	-	-	-	-	-	-	-	-	-	-	-
Agromyces	-	-	-	0.05	-	-	-	0.07	-	-	-	-	-	-	-	-	-	-
Lacunisphaera	0.07	-	-	0.05	-	-	-	-	-	-	-	-	-	-	-	-	-	-
Luteimonas	-	-	-	0.11	-	-	-	-	-	-	-	-	-	-	-	-	-	-
Bdellovibrio	0.10	-	-	-	-	-	-	-	-	-	-	-	-	-	-	-	-	-
Gaiella	0.03	-	-	0.07	-	-	-	-	-	-	-	-	-	-	-	-	-	-
Candidatus_*Koribacter*	-	-	-	-	-	0.10	-	-	-	-	-	-	-	-	-	-	-	-
Candidatus_*Xiphinematobacter*	0.05	-	-	0.02	-	-	-	-	-	0.03	-	-	-	-	-	-	-	-
Saccharothrix	-	-	-	0.10	-	-	-	-	-	-	-	-	-	-	-	-	-	-
Singulisphaera	-	-	-	0.09	-	-	-	-	-	-	-	-	-	-	-	-	-	-
Cryptosporangium	0.06	-	-	0.03	-	-	-	-	-	-	-	-	-	-	-	-	-	-

Luteibacter	-	-	-	0.09	-	-		-	-	-	-	-	-		-	-	-	-	-	-
Herbaspirillum	0.09	-	-	-	-	-		-	-	-	-	-	-		-	-	-	-	-	-
Ideonella	0.09	-	-	-	-	-		-	-	-	-	-	-		-	-	-	-	-	-
Actinoallomurus	-	-	-	0.08	-	-		-	-	-	-	-	-		-	-	-	-	-	-
Nitrospira	0.03	-	-	0.02	-	-		-	-	-	-	-	-		-	-	-	0.03	-	-
Stenotrophobacter	0.03	-	-	-	-	-		-	-	-	-	-	-		-	-	-	-	0.05	-
Sphingopyxis	0.02	-	-	0.05	-	-		-	-	-	-	-	-		-	-	-	-	-	-
Aquisphaera	-	-	-	0.07	-	-		-	-	-	-	-	-		-	-	-	-	-	-
Flavitalea	0.06	-	-	-	-	-		-	-	-	-	-	-		-	-	-	-	-	-
Phaselicystis	0.02	-	-	0.03	-	-		-	-	-	-	-	-		-	-	-	-	-	-
Paenibacillus	0.02	-	-	-	-	-		-	-	-	-	-	-		-	-	-	-	-	-
Pirellula	0.02	-	-	0.03	-	-		-	-	-	-	-	-		-	-	-	-	-	-
Nevskia	-	-	-	-	-	-		-	-	-	-	-	0.03		-	-	-	-	-	-
*Clostridium*_sensu_stricto	-	-	-	-	-	-		0.03	-	-	-	-	-		-	-	-	-	-	-
Pseudarthrobacter	0.03	-	-	-	-	-		-	-	-	-	-	-		-	-	-	-	-	-
Altererythrobacter	0.03	-	-	-	-	-		-	-	-	-	-	-		-	-	-	-	-	-
Rhizocola	-	-	-	0.02	-	-		-	-	-	-	-	-		-	-	-	-	-	-
Romboutsia	-	-	-	0.02	-	-		-	-	-	-	-	-		-	-	-	-	-	-
Adhaeribacter	-	-	-	0.01	-	-		-	-	-	-	-	-		-	-	-	-	-	-
Rubrobacter	-	-	-	0.01	-	-		-	-	-	-	-	-		-	-	-	-	-	-

Table S6. Relative abundance (%) of bacteria identified at the phylum, class, family and/or order level and unassigned genera present in the microbiome of roots of soybean non-inoculated (control), inoculated with *Bradyrhizobium* spp., or co-inoculated adding *Azospirillum brasilense*.

Genus	Soybean Roots																	
	Control						Inoculated						Co-inoculated					
	R1	R2	R3	R4	R5	R6	R1	R2	R3	R4	R5	R6	R1	R2	R3	R4	R5	R6
UnC Chitinophagaceae	1.81	1.84	2.34	1.37	2.38	1.27	1.49	1.85	1.99	1.79	2.71	1.39	1.30	1.84	1.42	2.65	1.31	1.29
UnC Gammaproteobacteria I.S.	1.27	1.72	1.05	1.12	1.20	1.43	0.91	1.17	1.17	1.29	1.83	1.53	0.76	1.09	0.98	1.46	1.19	0.93
UnC Saccharimonadales	1.25	-	-	0.72	0.30	-	-	-	0.93	0.52	1.45	0.17	0.76	5.61	-	0.37	0.67	1.45
UnC Xanthobacteraceae	0.66	0.89	0.94	1.04	1.04	0.66	0.75	1.60	0.35	1.27	0.37	-	0.52	0.47	-	0.82	0.44	0.46
UnC Micropepsaceae	0.55	-	0.84	0.64	0.59	0.79	0.73	0.64	0.61	0.54	0.54	1.25	0.33	0.53	0.52	0.51	0.24	0.41
UnC Burkholderiaceae	0.65	-	0.73	0.36	0.27	-	-	-	0.32	-	-	-	0.34	0.58	-	-	0.40	0.53
UnC Acidobacteriia	0.32	0.30	0.26	0.31	0.07	-	0.22	0.37	0.16	0.17	0.40	0.20	0.09	0.09	0.14	0.18	0.09	-
UnC Acidobacteriales	0.50	-	0.20	0.43	0.17	0.07	0.32	0.20	0.24	0.11	0.44	-	0.04	-	0.10	0.16	0.08	0.10
UnC Acidobacteria	0.70	0.58	0.10	0.83	-	-	0.22	0.11	-	0.17	0.04	-	0.06	-	0.13	-	0.05	-
UnC Acidobacteriaceae	0.02	-	-	-	-	-	-	-	-	-	-	-	-	-	-	-	-	-
UnC Myxococcales	0.16	-	0.08	0.19	0.09	0.04	-	-	0.10	0.01	0.15	-	-	0.04	-	0.01	0.04	-
UnC Tepidisphaerales	0.21	-	0.09	0.16	-	-	-	0.08	-	-	-	0.07	-	0.06	-	-	0.04	-
UnC Pyrinomonadaceae	0.21	-	-	0.21	-	-	-	-	-	0.22	-	-	-	-	-	-	-	-
UnC Acidimicrobiia	0.15	-	-	0.30	-	-	-	0.04	-	-	0.04	-	-	-	-	-	0.04	0.04
UnC Magnetospirillaceae	-	-	-	-	-	0.53	-	-	-	-	-	-	-	-	-	-	-	-
UnC Gaiellales	0.21	-	-	0.32	-	-	-	-	-	-	-	-	-	-	-	-	-	-
UnC Sphingomonadaceae	0.16	-	-	0.19	-	-	-	-	-	-	-	-	-	-	-	-	-	-
UnC Pedosphaeraceae	0.05	-	0.07	0.08	-	-	-	0.02	-	-	0.07	-	-	-	-	-	-	-
UnC Methyloligellaceae	0.09	-	-	0.16	-	-	-	-	-	-	-	-	-	-	-	-	0.04	-
UnC Solirubrobacterales	0.02	-	-	0.25	-	-	-	-	-	-	-	-	-	-	-	-	-	-
UnC Steroidobacteraceae	0.06	-	-	0.08	-	-	-	-	-	-	-	-	-	-	0.11	-	-	-
UnC Deltaproteobacteria	0.02	-	-	0.02	-	-	-	0.15	-	-	-	-	0.04	-	-	-	-	-
UnC Chloroflexi	0.07	-	-	0.15	-	-	-	-	-	-	-	-	-	-	-	-	-	-
UnC Micromonosporaceae	0.09	-	-	0.13	-	-	-	-	-	-	-	-	-	-	-	-	-	-
UnC Blastocatellaceae	0.03	-	-	0.07	-	-	-	-	-	0.03	-	-	-	-	0.07	-	-	-
UnC Ilumatobacteraceae	0.10	-	-	0.04	-	-	-	-	-	-	-	-	-	-	-	-	-	-

UnC Alphaproteobacteria	0.05	-	-	0.08	-	-	-	-	-	-	-	-	-	-	-	-	-	-
UnC Fimbriimonadaceae	0.12	-	-	-	-	-	-	-	-	-	-	-	-	-	-	-	-	-
UnC Betaproteobacteriales	-	-	-	-	-	-	-	-	0.07	-	-	-	-	-	-	-	0.04	-
UnC Rokubacteria	0.06	-	-	0.05	-	-	-	-	-	-	-	-	-	-	-	-	-	-
UnC Caulobacteraceae	0.06	-	-	0.05	-	-	-	-	-	-	-	-	-	-	-	-	-	-
UnC Frankiales	-	-	-	0.10	-	-	-	-	-	-	-	-	-	-	-	-	-	-
UnC Acetobacteraceae	-	-	-	0.10	-	-	-	-	-	-	-	-	-	-	-	-	-	-
UnC Gemmatimonadaceae	0.02	-	-	0.08	-	-	-	-	-	-	-	-	-	-	-	-	-	-
UnC Microscillaceae	0.05	-	-	0.03	-	-	-	-	-	-	-	-	-	-	-	-	-	-
UnC Rhodospirillaceae	0.04	-	-	0.04	-	-	-	-	-	-	-	-	-	-	-	-	-	-
UnC Holophagae	0.04	-	-	0.04	-	-	-	-	-	-	-	-	-	-	-	-	-	-
UnC Microtrichales	0.07	-	-	-	-	-	-	-	-	-	-	-	-	-	-	-	-	-
UnC Rhizobiales	0.02	-	0.02	0.01	-	-	-	-	-	-	-	-	-	0.02	-	-	-	-
UnC Hyphomonadaceae	0.03	-	-	-	-	-	-	0.02	-	-	-	-	-	-	-	-	-	-
UnC Blastocatellia	0.05	-	-	-	-	-	-	-	-	-	-	-	-	-	-	-	-	-
UnC Elsterales	-	-	-	0.05	-	-	-	-	-	-	-	-	-	-	-	-	-	-
UnC Gemmataceae	-	-	-	0.04	-	-	-	-	-	-	-	-	-	-	-	-	-	-
UnC Planctomycetales	-	-	-	0.04	-	-	-	-	-	-	-	-	-	-	-	-	-	-
UnC Gammaproteobacteria	-	-	-	0.02	-	-	-	-	-	-	-	-	-	-	-	-	-	-
UnC Sandaracinaceae	-	-	-	0.02	-	-	-	-	-	-	-	-	-	-	-	-	-	-
UnC Actinobacteria	0.01	-	-	0.02	-	-	-	-	-	-	-	-	-	-	-	-	-	-
UnC Pirellulaceae	-	-	-	0.02	-	-	-	-	-	-	-	-	-	-	-	-	-	-
UnC Rubinisphaeraceae	-	-	-	0.02	-	-	-	-	-	-	-	-	-	-	-	-	-	-
UnC Dehalococcoidia	-	-	-	0.02	-	-	-	-	-	-	-	-	-	-	-	-	-	-
UnC Nitrosomonadaceae	-	-	-	0.01	-	-	-	-	-	-	-	-	-	-	-	-	-	-
UnC Azospirillales	0.01	-	-	-	-	-	-	-	-	-	-	-	-	-	-	-	-	-
Unassigned	14.90	14.82	8.82	10.44	12.37	19.33	9.93	13.73	14.30	14.30	16.20	16.00	11.16	15.04	12.06	14.00	14.58	12.50

Table S7. Relative abundance (%) of the genera present in the microbiome of nodules of common bean non-inoculated (control), inoculated with *Rhizobium tropici*, or co-inoculated adding *Azospirillum brasilense*.

Genus	Common Bean Nodules																	
	Control						Inoculated						Co-inoculated					
	**R1**	**R2**	**R3**	**R4**	**R5**	**R6**	**R1**	**R2**	**R3**	**R4**	**R5**	**R6**	**R1**	**R2**	**R3**	**R4**	**R5**	**R6**
Allorhizobium-Neorhizobium-Pararhizobium-Rhizobium	90.51	95.57	89.67	94.18	95.83	81.51	92.95	89.69	89.29	91.03	92.68	86.75	94.99	95.79		88.46	89.64	96.51
Asticcacaulis	0.22	0.16	0.17	0.12	0.15	0.62	0.07	-	0.03	-	0.07	0.07	0.08	0.06	-	0.04	-	-
Bacillus	-	-	-	-	0.03	0.03	-	-	0.01	0.02	0.02	0.04	-	0.01	-	-	-	0.02
Bradyrhizobium	-	0.06	0.14	-	0.09	0.21	0.06	-	-	-	-	0.14	0.07	0.07	-	0.05	-	0.08
Chitinophaga	0.04	0.02	-	0.02	-	0.05	-	-	-	-	0.04	0.08	-	0.02	-	-	0.02	0.02
Devosia	0.08	0.03	0.08	0.03	0.06	0.20	-	-	0.03	0.07	0.07	0.12	-	-	-	0.07	0.03	-
Dyadobacter	0.16	0.15	0.26	0.11	0.15	0.45	0.08	0.20	0.13	0.20	0.19	0.51	0.05	0.08	-	0.17	0.18	0.07
Methylophilus	0.32	0.14	0.20	0.10	0.15	0.46	0.08	-	0.06	0.12	0.06	0.17	0.07	0.07	-	0.12	0.09	0.05
Niastella	-	0.06	-	-	-	-	-	-	-	0.01	-	0.03	-	-	-	-	-	0.06
Novosphingobium	0.35	0.09	0.15	0.11	0.15	0.34	0.06		0.05	0.09	0.13	0.16	0.05	-	-	0.14	0.06	0.11
Sphingomonas	0.25	0.11	0.23	0.09	0.11	0.38	0.07	0.13	-	0.08	0.09	0.13	-	0.07	-	0.08	0.07	-
Paenarthrobacter	0.03	0.03	-	-	-	0.05	-	-	-	0.03	0.04	0.08	-	-	-	-	-	0.04
Methylibium	0.09	0.07	0.12	-	0.03	0.09	0.05	0.05		0.05	0.07	0.11	0.03	0.04	-	0.06	-	0.05
Fluviicola	0.06	0.01	-	0.03	-	0.22	-	0.02	0.01	-	-	-	0.02	0.02	-	-	-	-
Emticicia	0.06	0.05	-	0.02	-	0.20	0.04	-	-	-	0.03	0.08	-	-	-	-	0.02	-
Streptomyces	-	-	0.11	-	-	0.28	0.06	0.06	-	-	0.06		0.04	0.04	-	0.10	-	0.09
Phaselicystis	-	-	0.03	-	-	0.03	-	-	-	-	-	0.02	-	0.01	-	0.01	-	-
Brevundimonas	-	-	-	-	-	-	-	-	-	-	-	0.02	-	-	-	-	-	-
Hydrogenophaga	-	-	0.03	-	-	0.04	-	-	-	0.02	-	0.03	-	-	-	0.06	-	0.01
Caulobacter	0.05		0.07	-	-	-	0.02	0.04	-	-	-	-	-	-	-	-	-	-
Dongia	0.01	-	-	-	-	0.01	0.02	-	-	-	-	0.03	-	-	-	-	-	-
Flavisolibacter	0.05	-	-	-	-	-	-	-	-	-	-	-	-	-	-	-	-	-
Hyphomicrobium	-	-	-	-	-	0.06	-	-	-	-	-	-	-	0.02	-	-	-	0.01
Lysobacter	-	-	-	-	-	-	-	-	0.01	-	-	-	-	-	-	-	-	-
Massilia	-	-	-	-	-	-	-	-	-	-	-	-	0.02	0.04	-	-	-	0.04
Methylobacterium	0.01	-	-	-	-	-	-	-	-	-	-	-	-	-	-	-	-	-

Nocardioides	-	-	-	-	0.02	0.03		-	-	-	-	-	0.01		-	-	-	-	-	-
Nordella	-	-	-	-	-	0.02		-	-	-	-	-	0.02		-	-	-	-	-	-
Pelomonas	-	-	-	-	-	0.11		-	-	-	-	-	-		-	-	-	0.11	-	-
Phenylobacterium	-	-	-	-	-	0.04		-	-	-	-	-	0.06		-	-	-	-	-	-
Pseudolabrys	-	-	-	-	-	-		-	-	-	0.01	-	-		-	-	-	-	-	-
Rhizobacter	-	-	-	-	-	0.07		-	-	-	-	-	-		-	-	-	0.05	0.07	-
Xylophilus	-	-	-	-	-	0.07		-	-	-	-	0.05	0.05		-	-	-	-	-	-
Sorangium	-	-	-	-	-	-		-	-	-	-	0.01	-		-	-	-	-	-	-
Bosea	0.02	-	-	-	-	0.02		-	-	-	-	-	-		-	-	-	-	-	-
Haliangium	-	-	0.02	-	-	-		-	-	-	-	-	-		-	-	-	-	-	-
Luteolibacter	-	-	0.02	-	-	-		-	-	-	-	-	-		-	-	-	-	-	-
Bdellovibrio	-	-	-	-	-	0.02		-	-	-	-	-	-		-	-	-	-	-	-
Pseudarthrobacter	-	-	-	-	-	-		-	-	-	-	-	-		-	-	-	0.04	-	-
Chryseolinea	-	-	-	-	-	-		-	-	-	0.01	-	-		-	0.01	-	0.01	-	-
Ohtaekwangia	-	-	-	-	-	0.03		-	-	-	-	-	-		-	-	-	-	-	-
Rubellimicrobium	-	-	-	-	-	-		-	-	-	-	-	0.01		-	-	-	-	-	-
Lapillicoccus	-	-	-	-	-	0.01		-	-	-	-	-	-		-	-	-	-	-	-
Microbispora	-	-	-	-	-	-		-	-	-	-	0.01	-		-	-	-	-	-	-
Terribacillus	-	-	-	-	-	-		-	-	-	-	0.003	-		-	-	-	-	-	-
Phycicoccus	-	-	-	-	-	-		-	-	0.005	-	-	-		-	-	-	-	-	-
Sphaerisporangium	-	-	-	-	-	-		-	-	-	-	-	-		-	0.003	-	-	-	-
Sphingobacterium	-	-	-	-	-	-		-	-	-	-	-	-		0.005	-	-	-	-	-
Dyella	-	-	-	-	-	-		-	-	-	-	-	-		0.005	-	-	-	-	-

Table S8. Relative abundance (%) of bacteria identified at the phylum, class, family and/or order and unassigned genera in the microbiome of nodules of common bean non-inoculated (control), inoculated with *Rhizobium tropici*, or co-inoculated adding *Azospirillum brasilense*.

**Genus**	**Common Bean Nodules**																	
	**Control**						**Inoculated**						**Co-inoculated**					
	**R1**	**R2**	**R3**	**R4**	**R5**	**R6**	**R1**	**R2**	**R3**	**R4**	**R5**	**R6**	**R1**	**R2**	**R3**	**R4**	**R5**	**R6**
Unc_Gammaproteobacteria I.S.	-	0.01	-	0.01	-	-	-	-	-	-	-	0.01	-	-	-	-	-	0.01
Unc_Saccharimonadales	-	0.03	0.02	-	-	0.04	0.01	-	-	-	-	0.02	-	-	-	-	-	-
Unc_Sandaracinaceae			0.03	0.01		0.02	-	-	-	-	-	-	-	-	-	-	-	-
Unc_Oligoflexales	0.02	0.04	0.03			0.07	0.02	0.05	-	-	0.01	0.06	-	-	-	0.02	-	-
Unc_Microscillaceae	-	-	-	-	-	-	-	-	-	-	-	-	-	-	-	-	-	0.01
Unc_Myxococcales	-	-	-	-	-	0.02	-	-	-	-	-	-	-	-	-	-	-	-
Unassigned	0.56	0.09	0.69	0.17	0.32	0.86	0.06	-	0.11	0.18	0.28	0.66	0.15	0.13	-	0.51	0.12	0.20

Table S9. Relative abundance (%) of the genera present in the microbiome of roots of common bean non-inoculated (control), inoculated with *Rhizobium tropici*, or co-inoculated adding *Azospirillum brasilense*.

Genus	Common Bean Root																	
	Control						Inoculated						Co-inoculated					
	R1	R2	R3	R4	R5	R6	R1	R2	R3	R4	R5	R6	R1	R2	R3	R4	R5	R6
Allorhizobium-Neorhizobium-Pararhizobium-Rhizobium	9.65	11.23	21.81	12.04	16.77	9.42	25.67	24.29	13.08	18.98	26.70	25.76	24.29	26.02	16.23	18.48	31.37	30.69
Achromobacter	0.38	0.47	0.10	0.27	0.44	0.17	0.24	0.23	0.34	0.39	0.33	0.18	0.61	0.57	0.17	0.62	0.19	0.21
Aeromicrobium	0.20	0.40	0.34	0.30	0.41	0.27	0.22	0.30	0.46	0.47	0.52	0.26	0.27	0.18	0.38	0.33	0.43	0.30
Asticcacaulis	1.07	0.74	0.66	0.69	0.68	1.32	1.06	0.74	0.74	0.26	0.52	0.43	0.96	0.70	0.58	0.30	0.28	0.60
Bacillus	1.37	1.56	0.63	0.93	1.12	1.38	1.72	1.25	1.26	1.47	1.80	1.01	2.00	1.39	0.99	1.11	1.17	0.97
Bradyrhizobium	3.73	3.27	3.43	3.15	1.70	2.54	2.23	4.09	3.65	3.39	2.75	3.91	3.55	2.59	3.30	2.93	2.60	2.90
Burkholderia-Caballeronia-Paraburkholderia	1.28	1.32	0.12	1.48	0.79	0.89	0.87	0.88	0.86	0.95	0.29	0.14	0.94	0.85	0.96	0.76	0.15	0.25
Caulobacter	0.81	0.99	0.42	0.92	0.61	0.43	0.60	0.69	0.81	0.72	0.65	0.48	0.92	0.39	0.86	0.39	0.50	0.63
Chitinophaga	1.12	1.49	0.97	1.97	2.02	1.09	1.10	1.16	1.57	1.75	1.05	1.06	0.83	1.38	1.31	1.72	1.42	1.68
Devosia	1.70	3.09	1.93	3.04	2.33	2.05	1.29	2.15	2.96	2.59	2.62	2.52	2.06	1.67	2.11	2.18	1.89	1.79
Dongia	0.18	0.14	0.10	0.22	0.14	0.15	0.17	0.22	0.19	0.25	0.25	0.21	0.18	0.17	0.35	0.15	0.08	0.26
Dyadobacter	2.14	3.69	3.00	4.40	3.58	4.02	2.68	2.89	4.41	4.47	3.07	2.82	2.75	1.89	4.90	1.69	2.79	1.83
Dyella	0.17	0.17	0.15	0.26	0.47	0.10	0.28	0.18	0.17	0.30	0.24	0.14	0.29	0.12	0.17	0.26	0.16	0.16
Flavisolibacter	0.35	0.26	0.39	0.33	0.29	0.37	0.33	0.67	0.13	0.29	0.26	0.29	0.36	0.35	0.44	0.37	0.43	0.31
Lysobacter	0.38	0.46	0.23	0.52	0.36	0.34	0.28	0.29	0.59	0.55	0.38	0.13	0.26	0.36	0.27	0.39	0.41	0.35
Massilia	9.81	10.10	6.23	6.62	6.22	6.72	10.06	7.67	6.44	5.83	6.84	3.30	7.57	6.72	6.90	7.39	5.41	8.02
Methylobacterium	0.13	0.30	0.03	0.18	0.08	0.05	0.20	0.08	0.16	0.07	0.13	0.06	0.10	0.06	0.06	0.16	0.06	0.13
Methylophilus	0.46	0.50	0.76	1.12	0.92	0.63	0.98	0.74	0.56	0.93	0.31	0.57	0.46	0.65	0.83	0.52	0.61	0.44
Microvirga	0.44	0.53	0.27	0.27	0.25	0.36	0.22	0.37	0.40	0.28	0.47	0.18	0.28	0.25	0.14	0.43	0.32	0.28
Niastella	1.20	1.32	2.24	1.89	1.48	1.47	1.46	1.64	2.02	1.68	1.19	1.47	1.07	1.46	1.99	1.50	1.60	1.05
Novosphingobium	5.97	3.75	2.42	2.35	2.96	2.33	2.42	2.28	3.22	2.84	2.26	1.84	2.72	2.40	2.48	2.57	2.51	3.72
Paenibacillus	0.18	0.23	0.07	0.16	0.26	0.24	0.26	0.18	0.29	0.48	0.17	0.09	0.24	0.10	0.27	0.09	0.21	0.30
Phenylobacterium	0.77	0.59	0.55	0.48	0.28	0.48	0.53	0.50	0.60	0.54	0.47	0.42	0.64	0.69	0.54	0.88	0.54	0.82
Rhizobacter	0.69	0.32	1.65	0.86	0.70	1.86	0.39	0.67	1.20	0.72	0.34	1.28	0.26	0.36	1.00	1.64	0.60	0.47
Sphingomonas	3.63	4.69	2.59	3.19	2.65	2.36	2.67	2.77	3.16	3.04	2.41	2.40	2.13	1.93	2.08	2.57	2.62	2.53

Streptomyces	9.11	10.36	7.20	7.17	13.90	13.03		8.87	6.67	6.84	8.64	6.16	4.30		6.89	11.86	4.74	10.24	8.17	9.55
Xylophilus	1.02	0.79	1.01	0.53	0.87	1.13		0.52	0.59	0.60	1.05	0.85	1.01		0.52	0.45	0.83	0.72	0.70	1.01
Sphaerisporangium	0.20	0.32	0.20	0.19	0.17	0.21		0.36	0.21	0.21	0.20	0.20	0.25		0.27	0.25	0.12	0.28	0.28	0.25
Haliangium	0.37	0.40	0.55	0.73	0.43	0.48		0.44	0.46	0.15	0.34	0.37	0.45		0.53	0.78	0.40	0.64	0.50	0.47
Ideonella	0.38	0.99	0.20	0.27	0.45	0.42		0.60	0.43	0.67	0.25	0.40	0.30		0.19	0.29	0.66	0.60	0.47	0.44
Phaselicystis	0.17	0.18	0.36	0.21	0.19	0.24		0.31	0.27	0.16	0.34	0.34	0.30		0.25	0.15	0.21	0.29	0.23	0.20
Pseudarthrobacter	0.29	1.42	0.23	0.38	0.45	0.68		0.31	0.27	0.55	0.65	0.53	0.36		0.25	0.18	0.34	0.43	0.52	0.43
Altererythrobacter	0.29	0.37	0.23	0.21	0.06	0.28		0.36	0.24	0.14	0.41	0.20	0.16		0.47	0.27	0.21	0.15	0.36	0.44
Hydrogenophaga	0.33	0.67	0.45	0.93	0.92	0.60		0.38	0.42	0.54	0.54	0.41	0.56		0.54	0.60	0.64	1.54	1.19	0.40
Methylibium	0.15	0.37	0.67	0.41	0.35	0.46		0.27	0.32	0.45	0.51	0.57	0.34		0.26	0.44	0.59	0.57	0.33	0.54
Chryseolinea	0.30	0.24	0.32	0.44	0.35	0.54		0.30	0.28	0.43	0.35	0.37	0.33		0.29	0.28	0.35	0.34	0.44	0.20
Fluviicola	0.37	0.33	0.59	0.28	0.42	0.96		0.42	0.46	0.46	0.12	0.31	0.34		0.16	0.27	0.61	0.33	0.34	0.25
Tumebacillus	0.16	0.14	0.06	0.14	0.06	0.17		0.38	0.29	0.09	0.17	0.13	0.09		0.22	0.16	0.17	0.07	0.07	0.05
Mesorhizobium	0.20	0.34	0.24	0.49	0.27	0.21		0.14	0.24	0.24	0.24	0.31	0.24		-	0.15	0.27	0.13	0.19	-
Hyphomicrobium	0.23	0.18	0.26	0.19	0.27	-		0.32	0.36	0.33	0.19	0.27	0.33		0.39	0.27	0.18	0.37	-	0.21
Micromonospora	0.13	0.23	0.09	0.18	0.10	-		0.16	0.11	0.16	0.15	0.11	0.08		0.14	0.17	-	-	-	-
Nocardioides	0.25	0.48	0.13	0.41	0.31	0.35		0.13	0.22	0.31	0.29	0.27	-		0.07	0.17	0.12	0.23	0.19	0.21
Nordella	0.08	0.19	0.09	0.11	0.10	0.08		0.06	0.06	0.11	0.12	0.10	0.09		0.04	0.05	0.08	0.09	-	0.08
Paenarthrobacter	0.71	1.65	0.65	1.33	0.88	1.16		0.26	0.48	1.12	1.43	1.39	0.84		0.55	-	0.89	0.95	1.06	0.77
Pelomonas	0.26	0.17	0.18	-	-	0.36		-	0.22	0.32	0.25	0.25	0.24		0.13	0.27	0.15	1.28	0.63	-
Pseudoduganella	0.19	0.25	0.18	0.15	-	0.23		0.27	0.18	0.32	0.22	-	0.13		0.19	0.17	0.20	0.11	0.13	-
Pseudolabrys	0.16	0.18	0.19	0.17	0.16	-		0.14	0.16	0.13	0.15	0.17	0.18		0.25	0.11	-	0.13	-	-
Sorangium	0.10	0.18	-	0.15	0.12	0.13		0.17	0.21	0.14	0.16	0.25	-		0.09	-	0.04	-	0.18	0.26
Bosea	0.06	0.04	0.16	0.09	0.15	0.07		0.08	0.18	0.09	-	0.17	0.26		0.11	0.14	0.19	0.14	0.16	0.16
Luteolibacter	0.18	0.16	0.41	0.11	0.33	0.22		-	0.13	0.26	0.26	0.17	0.39		0.05	0.16	-	0.07	-	0.17
Bdellovibrio	0.23	-	0.19	0.04	0.08	0.23		0.08	0.08	0.07	-	0.03	0.02		0.09	0.13	0.18	0.09	0.06	0.08
Flavitalea	0.13	0.36	0.15	0.27	0.16	0.23		-	0.14	0.17	0.13	0.15	0.21		-	0.14	0.17	0.11	-	-
Caenimonas	-	0.54	0.38	0.36	0.17	-		-	0.36	0.33	-	0.43	0.22		0.31	0.30	0.37	0.41	-	0.47
Pajaroellobacter	0.40	0.11	0.18	0.11	0.09	0.13		0.10	-	0.16	0.13	0.14	0.14		0.12	-	0.14	-	0.07	-
Emticicia	0.10	0.12	0.35	0.29	0.16	0.36		0.20	0.23	0.26	0.16	0.20	0.25		0.15	0.16	0.35	0.11	0.17	-
Phycicoccus	-	0.12	0.14	0.20	0.12	0.15		-	-	0.17	0.12	0.15	0.19		-	-	0.11	0.08	0.18	-
Ferruginibacter	0.09	0.12	0.09	-	0.06	0.16		0.18	0.13	0.06	-	-	0.07		0.09	-	0.04	0.07	-	0.05
Agromyces	0.02	0.06	-	0.04	-	0.06		0.04	-	-	-	-	0.02		0.05	-	-	0.06	-	-

Amycolatopsis	-	0.06	-	-	0.03	0.04		0.07	-	-	-	0.03	0.05		-	0.05	0.02	-	0.06	0.05
Aridibacter	0.15	0.06	-	-	-	-		0.20	-	0.10	-	-	0.10		-	-	0.12	-	-	-
Candidatus *Udaeobacter*	0.11	0.16	-	-	0.03	0.10		0.15	0.06	-	0.03	-	0.03		0.04	0.07	0.01	-	-	-
Candidatus_*Xiphinematobacter*	0.02	0.02	-	0.01	-	-		0.03	-	0.01	-	-	-		-	-	-	-	-	-
Nitrospira	0.08	0.12	0.02	0.06	0.04	0.07		0.04	0.04	-	0.04	0.04	-		0.02	-	0.01	-	-	-
Stenotrophobacter	0.11	0.09	-	0.05	0.05	0.12		0.09	-	-	-	-	-		-	-	-	-	0.04	-
Steroidobacter	0.04	-	-	0.16	0.16	-		0.08	0.20	-	0.09	0.06	-		-	0.14	0.11	-	-	0.16
Arthrobacter	-	0.41	-	0.25	0.29	-		0.22	-	0.30	0.31	0.29	-		-	-	0.30	0.20	0.27	0.20
Pseudorhodoferax	0.18	0.46	-	0.06	-	0.14		0.09	0.08	0.16	0.06	-	0.15		-	0.07	0.09	-	0.28	0.18
Roseimicrobium	0.22	0.07	-	0.09	-	0.07		0.05	0.06	0.09	0.03	-	-		0.03	-	0.06	0.07	0.05	0.12
Labrys	-	-	-	-	0.04	0.07		-	0.11	-	-	-	-		-	0.07	0.08	0.06	0.09	
Brevundimonas	-	0.23	-	0.11	-	0.17		-	-	0.08	-	0.08	-		-	0.26	-	0.22	0.13	0.12
Bryobacter	-	0.16	-	-	-	-		-	-	-	-	-	-		-	-	-	0.02	-	-
Rhodoplanes	0.20	0.24	0.13	0.21	-	-		-	0.19	0.24	0.10	0.14	0.29		-	0.12	0.11	0.13	0.25	0.13
Rhodopseudomonas	-	0.09	-	-	-	-		-	0.07	-	-	0.14	-		-	-	-	-	-	-
Shinella	-	0.19	-	-	-	0.31		-	-	0.24	-	-	0.32		-	-	-	1.18	0.72	-
Ramlibacter	0.40	0.45	-	-	0.15	0.48		0.35	-	0.47	0.47	-	0.44		0.25	0.15	-	-	-	-
Acidovorax	-	-	-	-	-	0.84		-	-	0.14	0.13	0.41	0.24		-	-	0.19	0.47	-	-
*Ellin*6067	0.26	-	0.15	-	0.08	0.17		-	0.18	-	-	-	0.11		0.21	0.15	-	-	-	-
Erythrobacter	-	0.14	-	-	0.22	-		0.15	-	0.08	-	0.13	-		-	-	-	-	-	0.17
*Ellin*6055	-	0.66	-	-	0.23	-		0.38	-	0.20	-	-	-		0.19	0.22	-	-	-	0.33
Pseudoflavitalea	0.15	0.11	-	0.04	0.14	-		-	0.16	-	-	-	-		-	0.09	-	-	-	-
Noviherbaspirillum	-	0.20	-	-	-	0.14		-	-	-	-	-	0.13		-	0.07	0.15	0.11	-	-
Cellulosimicrobium	-	-	-	-	-	-		-	-	0.17	-	-	-		0.11	0.04	-	0.18	-	-
Niabella	0.02	-	0.07	0.03	0.08	0.02		-	-	0.05	0.08	-	0.07		0.04	-	-	0.01	-	-
Polaromonas		0.12	-	-	-	-		-	0.19	-	-	-	-		-	-	0.10	-	-	-
Cohnella	0.03	0.04	-	-	0.04	-		-	-	0.06	0.06	0.09	-		-	0.05	0.02	0.03	-	-
Ohtaekwangia	-	-	-	-	0.14	0.20		-	0.19	-	-	0.06	0.05		-	0.04	-	0.07	0.11	0.08
Taibaiella	-	-	0.03	-	-	0.06		-	-	0.05	0.17	-	0.06		-	-	-	-	-	-
Lacunisphaera	-	0.08	-	0.07	0.05	-		-	-	-	0.07	-	0.09		0.05	-	-	-	-	-
Luteimonas	0.08	-	0.16	-	-	-		-	0.16	0.14	-	0.16	0.26		-	0.13	-	0.23	-	-
Gaiella	0.02	0.05	-	-	-	-		-	-	-	-	-	-		-	-	-	-	-	-
Nonomuraea	0.08	-	-	-	-	-		0.10	-	-	-	-	-		-	0.07	-	0.10	-	-

Tahibacter	-	-	0.23	-	-	-		-	-	-	0.09	-	-		-	-	-	-	-	-
Rubellimicrobium	0.03	-	-	0.02	-	-		-	-	0.04	-	-	0.05		0.02	-	-	0.04		0.04
Terrimonas	-	-	-	0.04	-	-		-	-	-	0.07	-	0.05		-	-	0.09	-	-	-
Xenophilus	-	-	-	-	-	-				0.07	-	-	0.08		-	0.10	-	-	-	-
Opitutus	-	-	-	-	-	-		-	-	-	-	-	0.12		-	-	0.12	-	-	-
Chryseobacterium	-	-	-	-	-	-		-	-	-	-	-	0.26		-	-	-	-	-	-
Comamonas	-	-	0.68	-	0.20	-		-	0.25	0.55	-	-	0.81		0.16	-	0.34	-	-	-
Cupriavidus	-	0.23	-	0.08	-	-		-	-	-	0.05	-	-		-	-	0.10	-	-	-
Dactylosporangium	-	-	-	-	-	0.03		-	-	-	-	-	-		-	-	0.06	-	-	-
Flavobacterium	0.24	-	0.44	0.20	0.04	-		-	-	-	-	0.20	-		-	-	-	-	-	-
Lechevalieria	0.09	0.15	-	-	-	-		-	-	-	-	-	-		-	0.11	-	-	-	-
Leifsonia	-	0.09	-	-	0.04	0.06		-	-	-	-	-	-		-	-	-	0.16	-	-
Pseudomonas	-	0.29	-	-	0.10			-	0.06	-	-	-	-		-	-	-	-	-	0.35
Pseudonocardia	-	-	-	-	-	0.02		-	-	-	-	-	-		-	-	-	0.01	-	-
Pseudoxanthomonas	-	0.01	-	-	0.22	-		-	-	0.31	-	-	-		-	-	-	-	-	-
Ralstonia	0.09	0.09	-	-	-	0.07		-	-	-	-	-	-		-	0.07	0.18	0.30	0.23	-
Brucella	-	-	-	0.04	-	0.07		-	-	-	-	-	0.13		-	-	-	-	-	-
Lapillicoccus	-	0.05	-	-	-	-		-	-	-	-	-	-		-	-	-	-	-	-
Microbispora	-	-	-	-	-	-		-	-	-	-	-	-		-	0.04	-	-	-	-
Blastococcus	0.04	-	-	-	-	-		-	-	-	-	-	-		-	-	-	-	-	-
Archangium	-	-	-	-	-	-		-	-	-	-	-	-		0.03	-	-	-	-	-
Bauldia	-	-	-	-	-	-		-	-	-	-	-	-		-	-	-	-	-	0.01
Blastocatella	-	-	-	-	-	-		-	0.18	0.06	-	-	-		0.08	-	-	-	-	-
Terrabacter	0.14	0.17	-	-	-	-		0.14	-	-	-	-	-		0.10	0.10	-	-	-	-
Intrasporangium	-	-	-	-	0.13	0.13		-	-	-	0.13	0.09	-		-	-	-	0.11	-	0.19
Variovorax	-	0.18	-	0.09	-	-		-	-	0.14	-	-	-		-	-	-	-	-	
Mitsuaria	-	0.41	-	-	-	-		-	-	-	-	-	-		-	-	-	-	-	-

Table S10. Relative abundance (%) of bacteria identified at the phylum, class, family and/or order and unassigned genera in the microbiome of roots of common bean non-inoculated (control), inoculated with *Rhizobium tropici*, or co-inoculated adding *Azospirillum brasilense*.

Genus	Common Bean Root																	
	Control						Inoculated						Co-inculated					
	R1	R2	R3	R4	R5	R6	R1	R2	R3	R4	R5	R6	R1	R2	R3	R4	R5	R6
Unc Gammaproteobacteria_I.S.	1.00	0.77	0.45	1.11	0.63	0.72	1.10	0.74	0.58	0.85	0.45	0.67	0.75	0.49	0.52	0.35	0.33	0.33
Unc Chitinophagaceae	0.27	0.35	0.52	0.34	0.23	0.45	0.36	0.20	0.32	0.40	0.36	0.24	0.34	0.20	0.45	0.34	0.57	0.27
Unc Sandaracinaceae	0.43	0.49	0.78	1.05	0.51	0.62	0.43	0.54	0.61	0.67	0.74	0.85	0.73	0.42	0.83	0.91	0.75	0.44
Unc Oligoflexales	0.51	0.50	0.57	0.55	0.24	0.56	0.66	0.59	0.66	0.36	0.22	0.37	0.45	0.31	0.31	0.47	0.25	0.33
Unc Sphingomonadaceae	0.92	0.34	0.27	0.59	0.44	0.69	0.32	0.47	0.63	0.35	0.72	0.12	0.47	0.39	0.22	0.45	0.16	0.37
Unc Myxococcales	0.13	0.03	0.06	0.04	0.03	0.15	0.17	0.05	0.04	0.25	0.14	0.16	0.02	0.15	0.17	0.13	0.02	0.04
Unc Saccharimonadales	0.25	0.56	0.10	0.43	0.12	0.34	0.17	0.11	0.06	-	0.16	-	0.27	-	0.13	0.15	0.12	0.15
Unc Xanthobacteraceae	0.19	0.17	0.10	0.12	0.10	0.11	0.17	0.13	0.20	0.16	0.11	0.10	0.13	-	0.09	0.15	0.12	0.15
Unc Micropepsaceae	0.27	0.17	-	0.23	-	0.10	0.21	0.17	0.13	0.13	-	-	0.18	0.10	0.12	0.13	0.14	0.18
Unc Steroidobacteraceae	0.12	0.12	0.08	0.18	0.13	0.15	0.16	-	0.07	0.10	0.08	0.11	0.27	-	-	-	0.23	0.07
Unc Acidobacteria	0.14	0.40	-	0.19	0.13	0.35	0.26	0.09	0.03	0.05	-	0.02	-	0.05	0.05	-	0.09	-
Unc Microscillaceae	-	0.24	0.04	0.43	0.21	0.08	0.06	-	0.13	0.35	0.24	0.33	0.21	-	0.25	-	0.34	0.05
Unc Nitrosomonadaceae	0.12	0.04	0.09	-	-	0.04	0.15	0.15	-	0.09	-	0.10	0.04	-	0.04	0.06	0.04	-
Unc Acidimicrobiia	0.02	0.08	-	0.03	0.06	0.05	0.04	-	0.02	0.04	0.03	0.04	0.02	0.02	0.01	0.04	0.06	0.07
Unc Microbacteriaceae	-	-	-	0.15	0.10	-	0.26	-	0.26	0.19	0.21	-	0.22	0.10	-	-	-	-
Unc Acidobacteriales	0.04	-	-	0.03	-	-	0.13	-	0.03	-	0.02	-	0.04	-	-	-	0.03	-
Unc Fimbriimonadaceae	0.10	-	0.05	0.05	0.04	-	-	-	0.06	-	-	0.06	-	0.06	-	-	-	0.05
Unc Rubinisphaeraceae	-	0.07	0.05	0.17	0.06	-	-	-	0.12	0.04	-	-	0.04	0.04	0.07	-	0.04	-
Unc Tepidisphaerales	0.07	0.12	0.04	-	-	-	0.11	-	-	0.04	0.05	-	-	0.05	0.03	-	-	-
Unc Gaiellales	-	-	0.02	-	-	0.03	0.04	-	-	-	-	-	-	-	0.02	-	-	-
Unc Holophagae	-	0.05	0.10	-	-	-	0.20	-	-	-	-	-	-	-	-	-	-	-
Unc Gammaproteobacteria	-	-	-	-	-	0.08	0.08	-	0.04	-	-	-	-	-	-	0.04	-	0.04
Unc Methyloligellaceae	-	-	-	0.05	0.06	-	0.07	0.11	-	-	-	0.05	0.05	-	-	-	-	0.04
Unc Gemmatimonadaceae	0.07	0.07	0.06	-	-	0.02	0.04	0.03	0.02	-	0.01	0.01	-	-	-	-	-	-
Unc Betaproteobacteriales	0.05	0.09	-	-	-	-	-	-	-	-	-	0.03	-	-	-	0.02	-	-
Unc Solirubrobacterales	-	-	-	-	-	-	-	-	-	-	-	0.01	-	-	-	-	-	-

Unc Chloroflexi	0.04	-	-	-	-	-		0.03	-	-	-	-	-		-	-	-	-	-	-
Unc Pedosphaeraceae	0.02	0.04	-	-	0.02	-		-	-	-	-	-	-		-	-	-	-	-	-
Unc Hyphomonadaceae	-	-	-	-	-	-		-	-	-	-	-	-		0.03	-	-	-	-	-
Unc Burkholderiaceae	-	-	-	-	-	0.42		-	-	-	-	-	-		-	-	-	-	0.30	-
Unassigned	21.57	15.74	19.70	20.12	13.44	22.97		13.55	14.58	19.78	17.19	13.65	14.21		12.50	13.99	21.32	19.84	13.95	14.70

Table S11. Statistical analysis of alpha-diversity in the microbiomes considering the treatments (non-inoculated, inoculated with specific rhizobia and co-inoculated with *A. brasilense* in soybean and common bean), and the matrix (nodule and root microbiomes).

Anova Shannon

```
                 Df Sum Sq Mean Sq  F value   Pr(>F)    
Matriz            1 296.78  296.78 5005.489  < 2e-16 ***
Treatment         2   0.80    0.40    6.777  0.00212 ** 
Matriz:Treatment  2   0.51    0.26    4.329  0.01718 *  
Residuals        65   3.85    0.06                      
---
Signif. codes:  0 ‘***’ 0.001 ‘**’ 0.01 ‘*’ 0.05 ‘.’ 0.1 ‘ ’ 1
```

Tukey

Root:Control	4.5920802	a
Root:Inoculated	4.3157360	ab
Root:Co-inoculated	4.1319400	b
Nodule:Inoculated	0.2827224	c
Nodule:Control	0.2703665	c
Nodule:Co-inoculated	0.2149949	c

Anova InvSimpson

```
                 Df Sum Sq Mean Sq F value   Pr(>F)    
Matriz            1  22040   22040 200.865  < 2e-16 ***
Treatment         2   1527     763   6.958  0.00183 ** 
Matriz:Treatment  2   1469     734   6.693  0.00227 ** 
Residuals        65   7132     110                     
---
Signif. codes:  0 ‘***’ 0.001 ‘**’ 0.01 ‘*’ 0.05 ‘.’ 0.1 ‘ ’ 1
```

Tukey

	InvSimpson <dbl>	groups <chr>
Root:Control	48.322636	a
Root:Inoculated	34.612664	b
Root:Co-inoculated	26.187459	b
Nodule:Inoculated	1.209538	c
Nodule:Control	1.109237	c
Nodule:Co-inoculated	1.076225	c

Table S12. Statistical analysis of beta-diversity in the microbiomes considering the treatments (non-inoculated, inoculated with specific rhizobia and co-inoculated with *A. brasilense*) the host legumes (soybean and common bean), and the matrix (nodule and root microbiomes).

Permanova

	Df	SumOfSqs	R2	F	Pr(>F)	
Treatment	2	0.01361	0.00584	1.9662	0.0464	*
Treatment:Matriz	3	1.89648	0.81394	182.6826	0.0840	.
Treatment:Matriz:Legume	6	0.21575	0.09260	10.3915	0.1513	
Residual	59	0.20417	0.08762			
Total	70	2.33001	1.00000			

```
---
Signif. codes:  0 '***' 0.001 '**' 0.01 '*' 0.05 '.' 0.1 ' ' 1
```
